# Balance Assessment Using a Smartwatch Inertial Measurement Unit with Principal Component Analysis for Anatomical Calibration

**DOI:** 10.3390/s23104585

**Published:** 2023-05-09

**Authors:** Benjamin M. Presley, Jeffrey C. Sklar, Scott J. Hazelwood, Britta Berg-Johansen, Stephen M. Klisch

**Affiliations:** 1 Mechanical Engineering, College of Engineering, California Polytechnic State University, San Luis Obispo, CA 93407, USA; 2 Statistics, College of Science and Mathematics, California Polytechnic State University, San Luis Obispo, CA 93407, USA; 3 Biomedical Engineering, College of Engineering, California Polytechnic State University, San Luis Obispo, CA 93407, USA

**Keywords:** inertial measurement unit, coordinate system discovery, calibration, posturography, balance, biomechanics, wearables, principal component analysis, smartwatch

## Abstract

Balance assessment, or posturography, tracks and prevents health complications for a variety of groups with balance impairment, including the elderly population and patients with traumatic brain injury. Wearables can revolutionize state-of-the-art posturography methods, which have recently shifted focus to clinical validation of strictly positioned inertial measurement units (IMUs) as replacements for force-plate systems. Yet, modern anatomical calibration (i.e., sensor-to-segment alignment) methods have not been utilized in inertial-based posturography studies. Functional calibration methods can replace the need for strict placement of inertial measurement units, which may be tedious or confusing for certain users. In this study, balance-related metrics from a smartwatch IMU were tested against a strictly placed IMU after using a functional calibration method. The smartwatch and strictly placed IMUs were strongly correlated in clinically relevant posturography scores (r = 0.861–0.970, *p* < 0.001). Additionally, the smartwatch was able to detect significant variance (*p* < 0.001) between pose-type scores from the mediolateral (ML) acceleration data and anterior-posterior (AP) rotation data. With this calibration method, a large problem with inertial-based posturography has been addressed, and wearable, “at-home” balance-assessment technology is within possibility.

## 1. Introduction

Many biomechanical fields are innovating beyond laboratory-based optical motion capture systems and pursuing wearable sensors for motion characterization. One of these fields is posturography, or balance assessment, for which wearable inertial measurement units (IMUs) are being employed as alternatives to force-plate platforms. IMUs are an attractive alternative when force plates may be unavailable for routine testing. Balance assessment validates health programs that address injury, prevention, and/or long-term health. Assessment of balance is popular in clinical studies and household use, and devices to collect these measurements are rapidly being pursued in current research. Balance is an involuntary, cerebellar mechanism for maintaining upright posture. Recent studies have demonstrated that 30% of adults over the age of 65 fall each year, and 1 in 5 of these falls end in fatality [1]. Another at-risk population includes individuals with traumatic brain injury and Parkinson’s disease: 39–67% of patients with traumatic brain injury struggle with balance deficiency [2]. Additionally, studies on overweight adolescents are detecting gait and balance abnormalities that have significant correlations with early-onset arthritis [3,4]. These issues motivate development of a robust tool to assess balance conveniently and repeatedly. Furthermore, health programs that strive to mitigate these issues need a way to clinically validate progress.

Wearable IMUs are a promising solution for routine balance assessment if their setup can be made simpler. Previous non-wearable efforts for accessibility introduced tests such as visual balance assessments that predict falls and assess balance performance quickly—such as the Berg Balance Scale [5,6,7]. However, visual balance tests have been shown to have floor or ceiling effects (e.g., participants obtain the maximum score every time) and require trained personnel, and slight within-participant changes are only detectable when balance is rapidly changing (e.g., stroke recovery) [5]. Technological solutions also exist; the current gold-standard devices for quantitative measurement of balance are force plates, which can track center of pressure (COP) and ground reaction force vectors. However, they remain expensive and immobile compared with visual balance tests [1,8]. This has led to state-of-the-art research on IMUs, which can measure the three-dimensional gravity vector via a magnetometer, three-dimensional angular velocity data via a gyroscope, and three-dimensional acceleration data via an accelerometer. When attached to a human attempting to stand still, their performance can be quantified by how little the IMU measurements vary. Many studies agree that IMUs are capable of measuring balance [1,8,9,10,11].

Smartwatches with built-in IMUs push the frontier in wearable health technology. These are the most popular wearable devices with an expected 109.2 million purchases in 2023 [12]. Smartwatches have been marketed as a health tool but their utility for balance assessment remains unexplored. They were used in this study to explore functional calibration of an IMU for balance assessment.

Anatomical calibration is a popular sector of IMU research, but its state-of-the-art techniques have not been employed in IMU balance studies. Although it remains a goal for IMUs to increase the accessibility of balance assessment, in prior studies they were strictly placed so that their coordinate system was aligned with a meaningful coordinate system, which is often referred to as the global coordinate system—this procedure is known as assumed alignment [13]. Assumed alignment is a method for anatomical calibration, not to be confused with sensor calibration which involves the tuning of sensor magnitude. In this paper, calibration refers to anatomical calibration (i.e., sensor-to-segment alignment). Better anatomical calibration methods have been developed [13], but never applied to inertial posturography. There is a need to eliminate the strict initial placement procedure because it is a barrier to accessibility and ease-of-use. The human body’s coordinate system consists of the anteroposterior, mediolateral, and superior-inferior axes. According to a recent systematic review of 73 peer-reviewed studies on strictly placed inertial measurement units (SPIMUs), all referenced studies strictly placed the IMU so that the IMU’s coordinate system would match the global coordinate system [9]. The coordinate system may be important because specific poses can generate specific changes in motion data along each axis. For example, a recent study validating IMUs found that only medio-lateral motion data changed significantly between pose difficulties [11]. Of the four proven anatomical calibration methods [13,14], two are feasible specifically for posturography. Assumed alignment (i.e., strict placement of the IMU on a segment) and functional calibration (i.e., calibration from a prescribed maneuver or pose). Strict placement can lead to incorrect attachment to the global coordinate system. For example, 47 recent IMU balance-assessment studies had the IMUs strictly placed on the L5 disc region of the back [9]. Participants have different natural curvatures of the spine, which may introduce incorrect orientation of the sensor. This leaves functional calibration as a promising method.

The functional calibration algorithm employed in this study is based on principal component analysis (PCA). Two recent biomechanics studies have utilized a PCA-based calibration algorithm that allows an IMU to align itself with a single global axis based on movement known to be one-dimensional [15,16]. Other studies have also used a gravity vector to find a second reference axis, and then a cross product to find the third global axis [13,17]. This paper similarly reports the combination of a PCA maneuver, gravity vector, and cross product that aligns a smartwatch’s IMU in all three directions.

In addition, it must be stated that interpretation of sensor data in posturography has remained inconsistent. Even the parameters used to interpret force-plate data have remained controversial [9,18]. Posturography studies have typically used root-mean-square (RMS) to simplify sensor timeseries data to a single value, and this RMS value can act on COP velocity [8,19,20,21], COP position [19,20,21], and COP path length [18] parameters. The force-plate parameter used for this study was COP velocity, as this was recently shown to be the most reliable in young healthy adults [19], which is the population that comprises the sample for this study. IMU parameters used in previous studies have typically been RMS acceleration and RMS rotational velocity. These parameters are also typically isolated by direction: anterior-posterior (AP), mediolateral (ML), and sometimes superior-inferior (SI) [9]. Some studies are pursuing posturography scores that do not require alignment of the IMU at all—such as the 3D convex polyhedron score which shows significant score differences between pose types [22]. However, it is still most typical to isolate direction and report RMS posturography scores [9].

The hypotheses of this study were: (1) the magnetometer’s gravity vector and a PCA calibration maneuver can be used to align the smartwatch so its rotational velocity-based balance assessment correlates with a SPIMU’s rotational velocity-based balance assessment; and (2) the smartwatch, once calibrated, will be able to detect within-participant balance-assessment differences across three different balance poses that increase in difficulty.

## 2. Materials and Methods

Experimental protocols were approved by our Institutional Review Board for Human Subjects Research and were designed to minimize risk to human participants.

### 2.1. Participant Recruitment

In total, 18 participants (6 male, 12 female; ages 13–24; 5 youths and 13 adults) were recruited from our community. Individuals with lower extremity injuries or pain within the last 6 months were excluded from the study. No grouping of the participants was performed as this study is a comparison of a measurement device.

### 2.2. Balance Experiment

**Figure 1:**
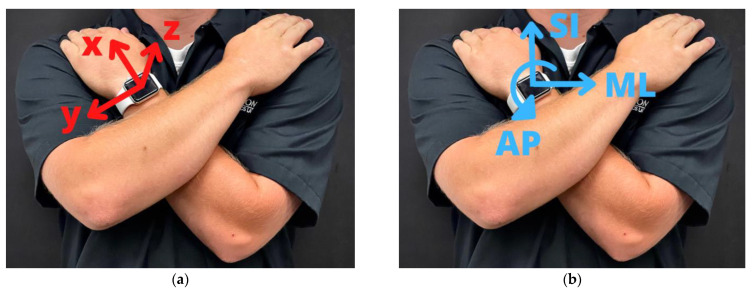
(**a**) Smartwatch’s inertial coordinate system, showing the directions in which the smartwatch records acceleration, rotation rate, and gravitational field. (**b**) Global coordinate system, which contains the anterior-posterior (AP) direction, mediolateral direction (ML), and superior-inferior (SI) direction.

The balance experiment was composed of three 30-second static poses that increased in difficulty. Three devices—a smartwatch, force plate, and SPIMU—were used to record data. Participants wore the smartwatch on their left wrist and were asked to hold onto their shoulders with each of their hands, as comfortably as possible, with the left arm under the right arm (Figure 1). This approach was chosen so that the smartwatch captured kinematic data similar to that of the trunk, which is a body segment that contains pertinent balance motion data [9], and to eliminate the need for extra equipment in an attempt to make the method easier to use in a real-world setting. The 30-second pose duration was chosen as that was the most common pose duration in previous studies [9]. Participants were instructed to practice the poses by either performing the entire experiment twice or performing each pose during a lab researcher’s demonstration.

Before each test, each participant performed three calibration maneuvers. Calibration maneuvers were used with a PCA-based algorithm which can identify the AP or ML axis within the inertial coordinate system. A calibration maneuver is a 1-dimensional maneuver (i.e., along 1 axis) in the global coordinate system. The first maneuver was the pounding of the chest: the participant lifts their arms off their chest, and—while still holding their arms in the crossed position—forcefully reinitiates the pose. The purpose is to generate a large, 1-dimensional acceleration in the anterior direction from the reaction force as the arms strike the chest. The second maneuver was 15-degree forward flexion of the trunk (i.e., bend forward and then back to normal stance). This is a 1-dimension maneuver measured by the gyroscope data about the ML axis. The third maneuver was 15-degree lateral bending of the trunk to the participant’s right-hand side (i.e., bend right and bend back to normal stance). This is a 1-dimensional maneuver measured by the gyroscope data about the AP axis. Some participants flexed more than the required 15 degrees as it was more natural for them to do so. The PCA-based algorithm described in Section 2.4 can identify the principal components of these maneuvers.

**Figure 2:**
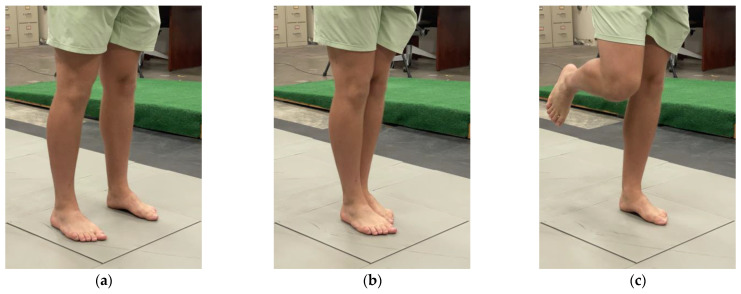
Images of different pose difficulties. (**a**) Both-legs stance; expected to be the least difficult. (**b**) Semi-tandem stance; expected to have moderate difficulty. (**c**) Single-leg stance; expected to be the most difficult.

Each static balance test consisted of one of three different poses of varying difficulty (Figure 2). The first static test was a standing test with both feet on a 502x502 mm force plate in a comfortable position. The second test was a semi-tandem stance with the dominant foot forward. The last test was a non-dominant single-leg stance. Research staff ensured all participants were able to comfortably stand in all poses in the center of the force plate. These poses were chosen as they were used in a long-established balance test developed by Berg et. al. in 1992 [7] and in recent inertial balance validation studies [8,11] on both healthy [7,11] and balance-deficient [7,8] participants. Only three poses were used, in order to increase statistical power by reducing the number of variable factors. All static tests were performed barefoot with eyes open. At the end of the test, the participant performed a small jump to create a data spike across each of the measurement devices for time synchronization. Synchronization was manually performed in MATLAB (MathWorks, Natick, MA, USA) by trimming the time series to 30 seconds, ending on the heel-strike spike. To ensure the heel-strike spike and calibration maneuvers would not affect the score, only the middle 24 seconds were used for the balance-assessment score and subsequent statistical analysis. Recording was started and stopped for each of the tests to make the trimming process easier.

Some participants moved their arms from the instructed pose to maintain balance. This marked a failed attempt, and they were asked to repeat the calibration maneuvers and pose. The static tests were expected to mitigate failed attempts while remaining difficult enough to generate meaningful changes in COP velocity and trunk acceleration.

### 2.3. Hardware Devices and Data Acquisition Software

The respective software interfaces of the smartwatch, force plate, and SPIMU were used to export the measured data and for post-processing with MATLAB. Post-processing involved trimming, running the smartwatch functional calibration algorithm, filtering, and generating the typical balance-assessment scores. 

The smartwatch used was an Apple Watch Series 3 (Apple, Cupertino, CA, USA). The participant wore this device on their left wrist, on the distal side. The smartwatch band was tightened enough so that it could not rotate on the wrist. The Apple Watch recorded acceleration, rotation rate, and the gravitational field at 100 Hz—which is its maximum sampling rate. Many studies have been successful at this sampling rate [9]. The smartwatch started, stopped, and exported data to a .csv file using software called SensorLog (Version 5.2, Bernd Thomas, Stuttgart, Germany), which runs on both an Apple Watch and iPhone and acts as a remote controller for starting, stopping, and exporting data. After performing calibration algorithms and time synchronization with other devices, a 3.5 Hz cutoff, zero-phase, 4th order Butterworth filter was applied [23,24].

The participant stood in the center of an AMTI AccuGait force plate (AMTI, Watertown, MA, USA) during each balance experiment so that their anterior-posterior axis was aligned with the force plate’s x-axis and their mediolateral axis was similarly aligned with the force plate’s y-axis. Cortex analysis software (Version 7.4.6, Motion Analysis Corporation, Rohnert Park, CA, USA) was used to start and stop data collection and to export the data to a .csv file. The force plate measured COP at 150 Hz in its x- and y-directions, which correspond to the AP and ML directions, respectively. During post-processing, the COP velocity was calculated via differentiation of COP position data. After carrying out time synchronization with other devices, the data was filtered with a 10 Hz cutoff, zero-phase, 4th order Butterworth filter [24].

A MetaMotions IMU (MMS, MetaMotions, CA, USA) was used as the SPIMU. The location of the SPIMU was on the L1 of the back, and the SPIMU was oriented so that its inertial axes were aligned with the global axes. The SPIMU’s x-axis was the negative global ML axis, the y-axis was the positive global SI axis, and the z-axis was the negative AP axis. The SPIMU was controlled with the MetaBase application (MMS, MetaMotions, CA, USA), through which data were started, stopped, and exported to a .csv file. The SPIMU recorded acceleration and rotation rate at 100 Hz and employed a 3.5 Hz cutoff, zero-phase, 4th order Butterworth filter. Filtering was applied after time synchronization of the data with other devices.

### 2.4. Calibration Algorithm

The PCA calibration algorithm is based on a principal component vector of acceleration or rotational velocity data during a calibration maneuver, a gravity vector from the magnetometer, and a cross-product of those two vectors. These vectors are “target” vectors—unit vectors that define the global coordinate system within the inertial coordinate system. There are three target vectors for each of the three global axes, and a dot product procedure was used to project inertial coordinate system data to the global axis target vectors so that the smartwatch can calculate balance-assessment measurements with respect to the global axes.

PCA is a dimensionality-reduction procedure that analyzes a set of multidimensional data and finds a principal component or a single vector that best describes the variance of that dataset. When PCA is utilized in three-dimensional (3D) motion timeseries data, it outputs the vector that best describes the movement of that time series. Using data from each of the calibration maneuvers described in Section 2.2, PCA calculated the principal direction of each calibration maneuver. As previously described, the calibration maneuver was a one-dimensional maneuver along a single axis in the global coordinate system. The other target vectors were found using the smartwatch’s built-in magnetometer and then a cross-product function between the first two vectors. All target vectors were scaled to unit vectors. Once the three target vectors were found within the inertial coordinate system, the entire timeseries dataset was projected onto these targets with the dot product.

Projection was computed for the entire time series of the data. Since the smartwatch was recording at 100 Hz, there were many projections to these discovered global target vectors. The target vectors stayed the same throughout the experiment and no dynamic tracking method was used. 

#### 2.4.1. Gravity Target Vector

The gravity vector points in the opposite direction of the human superior-inferior (SI) axis when the human torso is upright. Thus, a target vector was found by reading the smartwatch’s onboard magnetometer, which provides a unit gravity vector in the inertial coordinate system, and then calculating its negative vector. 

The magnetometer is always recording, so for this study, the gravity vector was averaged across the duration of the upright stance between the calibration maneuvers and the heel strike. The following MATLAB function was used to find the SI target vector, $v^{SI}$:

(1) $$v^{SI}=mean(-g_{t=posestart},-g_{t=posestart+1},\ldots ,-g_{t=poseend})$$

where g is the magnetometer’s gravity vector and mean is a function that calculates the mean gravity vector components across a time series of data. A variable in bold type in an equation indicates that the variable is a vector variable with 3 scalar components, while a non-bolded variable (used in the below equations) indicates a scalar component variable. After this target axis vector was found, the data in the inertial frame was projected onto it using:

(2) $${A_{SI}}_{i}={a_{x}}_{i}v_{x}^{SI}+a_{y}v_{y}^{SI}+a_{z}v_{z}^{SI}$$

(3) $${\Omega_{SI}}_{i}=\omega_{x}v_{x}^{SI}+\omega_{y}v_{y}^{SI}+\omega_{z}v_{z}^{SI}$$

where $A_{SI}$ is an acceleration vector in the super-inferior direction, $\Omega_{SI}$ is the rotation rate vector in the superior-inferior direction, lowercase variables (such as $a_{x}$ and $\omega_{x}$) represent acceleration and rotational velocity components, and subscripts indicate axes of the coordinate system. Specifically, x, y, and z represent the axes of the inertial coordinate system and AP, ML, and SI represent the axes of the global coordinate system.

The SI data were shared by the multiple calibration maneuver methods because they were obtained via the magnetometer. Additionally, this calibration alone allowed some classic IMU balance parameters, such as 2D magnitude (i.e., magnitude of AP + ML motion) to be calculated. The 2D magnitude was considered a “direction” and is assessed later in this study. These 2D magnitude values were solved with the equations below.

(4) $$A_{2D}=\left|a-(a\cdot v^{SI})v^{SI}\right|$$

(5) $$\Omega_{2D}=\left|\omega -(\omega \cdot v^{SI})v^{SI}\right|$$

#### 2.4.2. PCA Methods for the Forward Flexion Maneuver

**Figure 3:**
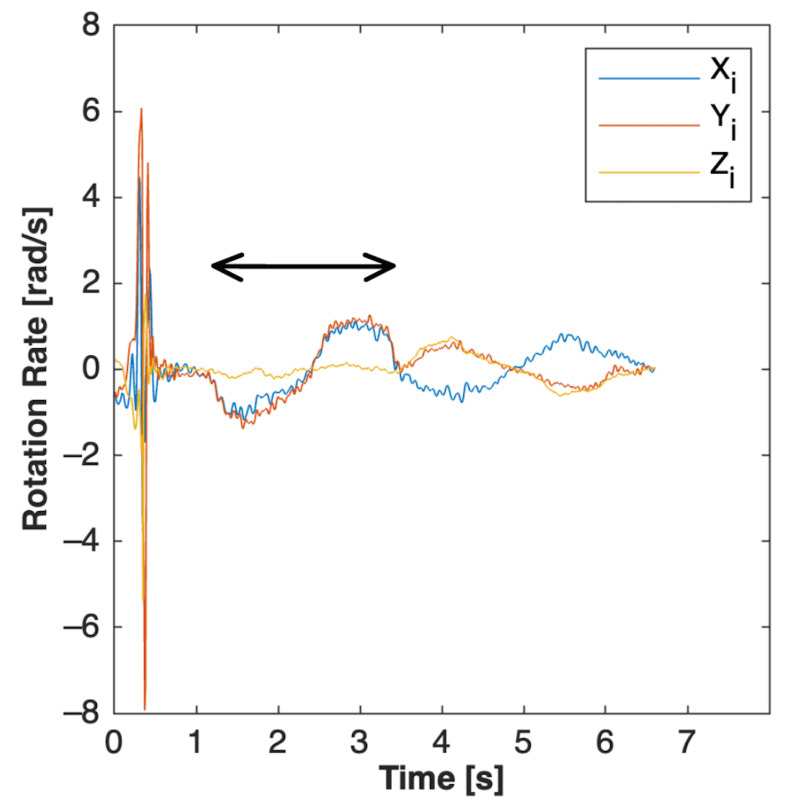
Manual selection of forward flexion (FF) calibration maneuver timeseries for input into PCA algorithm. The arrow bar above the data indicates the timeseries data that were identified as the forward flexion calibration maneuver. The noise on the left is from the chest-tap calibration maneuver. The sine wave to the right of the forward flexion maneuver is the lateral bending maneuver. The order of the calibration maneuvers was the same every time, so the researcher knew which maneuver was which.

PCA on the forward flexion (FF) calibration maneuver was employed to identify the ML axis. The calibration maneuver data were manually identified in the sensor’s timeseries data. The researchers selected a start and stop time for each calibration maneuver. An example is shown in Figure 3 below, for selection of the FF time series.

This data were then processed using a traditional PCA computation. First, the dataset from the calibration maneuver was centered. Then, the covariance matrix was calculated using:

(6) $$C=\left[\begin{array}{ccc} cov(\underline{\omega_{x_{c}}},\underline{\omega_{x_{c}}}) & cov(\underline{\omega_{x_{c}}},\underline{\omega_{y_{c}}}) & cov(\underline{\omega_{x_{c}}},\underline{\omega_{z_{c}}})\\ cov(\underline{\omega_{y_{c}}},\underline{\omega_{x_{c}}}) & cov(\underline{\omega_{y_{c}}},\underline{\omega_{y_{c}}}) & cov(\underline{\omega_{y_{c}}},\underline{\omega_{z_{c}}})\\ cov(\underline{\omega_{z_{c}}},\underline{\omega_{x_{c}}}) & cov(\underline{\omega_{z_{c}}},\underline{\omega_{y_{c}}}) & cov(\underline{\omega_{z_{c}}},\underline{\omega_{z_{c}}}) \end{array}\right]$$

where the underbar below a variable represents a chronological timeseries variable of the calibration maneuver and subscript c indicates a centered dataset. For example, $\underline{\omega_{x_{c}}}$ is a chronological list of all the centered, x-direction rotation rate magnitudes during the forward flexion maneuver. The cov function calculates the covariance between two sets of data:

(7) $$cov(\underline{x},\underline{y})=\frac{\sum \left({\underline{x}}_{i}-\bar{\underline{x}})({\underline{y}}_{i}-\underline{\bar{y}}\right)}{N}$$

where N is the number of elements in the dataset and ${\underline{x}}_{i}$ and ${\underline{y}}_{i}$ are components of the $\underline{x}$ and $\underline{y}$ dataset inputs, respectively. 

Next, the principal eigenvector $v_{PCA}^{ML}$ of the covariance matrix C was calculated and scaled to a unit vector using:

(8) $$v^{ML}=\frac{v_{PCA}^{ML}}{\left|v_{PCA}^{ML}\right|}$$

Here, the sign of the eigenvector was decided automatically by the algorithm. It is not possible for the eigenvector to predict the positive or negative ML direction—only the axis itself. Since the forward flexion began with positive rotation, the algorithm checked whether the first peak of the forward flexion maneuver’s timeseries data $\omega_{peak_{1}}$ was positive or negative and multiplied the value by one or negative one, respectively. This was done with the value of the dot product between $v^{ML}$ and ${\omega_{peak}}_{1}$ obtained using:

(9) $$c_{sign}=v^{ML}\cdot \omega_{peak_{1}}$$

(10) $$v^{ML}=v^{ML}\cdot \frac{c_{sign}}{\left|C_{sign}\right|}$$

where $c_{sign}$ is a coefficient that holds the sign corrector for the target axis.

Next, the inertial data were projected to the global ML axis using:

(11) $${A_{ML}}_{i}={a_{x}}_{i}v_{x}^{ML}+{a_{y}}_{i}v_{y}^{ML}+{a_{z}}_{i}v_{z}^{ML}$$

(12) $${\Omega_{ML}}_{i}={\omega_{x}}_{i}v_{x}^{ML}+{\omega_{y}}_{i}v_{y}^{ML}+{\omega_{z}}_{i}v_{z}^{ML}$$

Since there were two other calibration maneuvers, these data were saved and marked as ML axis data found with forward flexion calibration.

#### 2.4.3. PCA Methods for the Lateral Bending Maneuver

The lateral bending maneuver created a substantial, principal rotation about the AP axis. The mathematics were the same as the forward flexion maneuver; however, the covariance matrix was given rotational velocity data for the period of the lateral bending maneuver, and the principal eigenvector of this covariance matrix was theoretically aligned with the global AP axis. This vector was then scaled to a unit vector and its sign was verified in the same manner as before. The uncalibrated data were projected to the newly found global axis target using:

(13) $${A_{AP}}_{i}={a_{x}}_{i}v_{x}^{AP}+{a_{y}}_{i}v_{y}^{AP}+{a_{z}}_{i}v_{z}^{AP}$$

(14) $${\Omega_{AP}}_{i}={\omega_{x}}_{i}v_{x}^{AP}+{\omega_{y}}_{i}v_{y}^{AP}+{\omega_{z}}_{i}v_{z}^{AP}$$

Since there were two other calibration maneuvers, these data were saved and marked as AP axis data found with lateral bending calibration.

#### 2.4.4. PCA Methods for the Chest Tap Maneuver

The chest-tap maneuver created a substantial, principal acceleration on the AP axis. The mathematics were the same as previous maneuvers; however, the covariance matrix was given acceleration data for the period of the chest tap maneuver, and the principal eigenvector of this covariance matrix was theoretically aligned with the global AP axis. This variable was then scaled to a unit vector in the same manner as before.

Its sign, however, was assigned more rigidly than before. There were 3 different peaks of acceleration along the AP axis during the chest tap, and the largest peak was highly dependent on how forceful the participant was when performing the chest-tap maneuver. Since the smartwatch’s screen was facing outwards in the AP direction, the smartwatch’s z-axis lay in the positive AP axis. So, the algorithm checked whether the dot product between the target vector $v^{AP}$ and smartwatch’s z-axis $e^{z}$ was positive, and it flipped the sign if it was not.

(15) $$c_{sign}=v^{AP}\cdot e^{z}$$

(16) $$v^{AP}=v^{AP}\cdot \frac{c_{sign}}{\left|C_{sign}\right|}$$

The uncalibrated data were projected to the newly found global axis target with the dot product (see Equations (13) and (14)). Since there were two other calibration maneuvers, these data were saved and marked as AP axis data found with chest-tap calibration.

#### 2.4.5. Cross-Product Utilization

With a gravity vector and a PCA maneuver, the smartwatch found 2 global axis targets. The cross product found the third and final axis. The global axes were fully defined three times—once for each maneuver. Table 1 below shows how each calibration maneuver method utilized the cross product to find the final axis.

The cross product was verified to be a unit vector, and then inertial data were projected onto the final target vector with the dot product. The data were saved with the calibration maneuver’s directional counterpart; the full datasets (those with AP, ML, and SI motion data) were grouped based on the PCA calibration maneuver that was used to discover one of the axes. To clarify, the SI data were the same across all maneuvers because they all shared the magnetometer’s target vector (see Section 2.4.1).

### 2.5. Statistical Methods

The statistical design of this study was conducted to determine (1) whether there was a significant correlation between rotation-based posturography scores from the smartwatch’s IMU and the SPIMU, (2) whether there was a significant correlation between force-plate COP velocity and smartwatch acceleration posturography scores, and (3) whether the smartwatch’s posturography scores could detect within-participant differences between pose-type conditions. The default significance level for the statistical analysis was α = 0.05.

Pearson correlations were used to address the first and second objectives. Correlation coefficients were tiered as follows: 0.1 represents a weak correlation, 0.3 a moderate correlation, and 0.5 a strong correlation. This hierarchy is consistent with a recent balance study [8] that followed behavioral science analysis methods from Cohen et. al. [25]. Repeated measures analysis of variance (RMANOVA) was used to investigate the third objective. Each device, direction, and metric were tested for detection of variation in the mean scores by pose type. Further detailed statistical methods for each of the 3 objectives are described below.

#### 2.5.1. Smartwatch versus SPIMU Correlation Design

A two-tailed Pearson correlation test was performed between the scores of the smartwatch and SPIMU devices. The score parameter used was the RMS rotation rate isolated in each direction. This value was selected because the smartwatch and SPIMU were on different sides of the participant’s trunk, so rotational velocity was likely to be a more similar comparison than acceleration. Correlations were performed independently in the AP, ML, and 2D directions (see Section 2.4.1, Equations (4) and (5), for discussion of 2D “direction”). Across 18 participants, 84 distinctive poses were recorded by the smartwatch and SPIMU simultaneously. Each device provided a score for that distinctive pose (both the smartwatch and SPIMU generated 84 data points to use in the correlation study). Furthermore, the smartwatch performed 3 different calibration maneuvers, so the smartwatch’s inertial dataset was transformed onto the global coordinate system in 3 different ways. Each calibration method had its own score and interpretation of the balance performance of each distinctive pose.

#### 2.5.2. Smartwatch versus Force Plate Correlation Design

A two-tailed Pearson correlation test was performed between the scores of the smartwatch and force-plate devices. The score parameter for the smartwatch was the RMS acceleration isolated in each direction. The score parameter for the force plate was the RMS COP velocity isolation in each direction, in line with a recent study [8]. Correlations were performed independently in the AP, ML, and 2D directions. Across 18 participants, 84 distinctive poses were recorded by the smartwatch and force plate simultaneously. Each device provided a score for that distinctive pose (both the smartwatch and force plate generated 84 data points to use in the correlation study). They were expected to be positively correlated to one another, based on [8]. Furthermore, the smartwatch performed 3 different calibration maneuvers, so the smartwatch’s inertial dataset was transformed onto the global coordinate system 3 different ways. Each calibration method had its own score and provided an interpretation of the balance performance of each distinctive pose. 

#### 2.5.3. Smartwatch Repeated-Measures Analysis of Variance across Pose Types

RMANOVA was completed to test whether the smartwatch could detect significant variation in posturography scores across three increasingly difficult pose types. Since it is still debated which IMU posturography scores are relevant, RMS acceleration and RMS rotational velocity were both analyzed. The RMANOVA tests were also run for the SPIMU and force plate for comparison. Additionally, each directional score was tested: AP, ML, 2D, and 3D. The 3D “direction” is the combined magnitude of the AP, ML, and SI motion vectors. To reduce risk of type 1 error from multiple testing, only the calibration maneuver with the highest SPIMU correlation was used. For further protection against statistical type 1 error, a Bonferroni correction was used. The corrected, experimental-wise significance level $\alpha_{e}$ was used for the assessment of the RMANOVA results.

(17) $$\alpha =0.05,n_{tests}=19$$

(18) $$\alpha_{e}=\frac{\alpha }{n_{tests}}=0.0026$$

Data were analyzed for the 18 participants who had completed three poses of varying difficulty (N = 18). If the participant completed some of the pose types more than once, the most recent instance of that pose type was used for scoring in the RMANOVA study. Sphericity is an assumption for RMANOVA studies. To assume sphericity is to assume that the variances of the differences between the RMS measurements for the different poses are identical—which is likely to be a false assumption. In instances where this occurred, a Greenhouse–Geisser correction was applied to the degrees of freedom (DOF) for the test [26,27].

## 3. Results

### 3.1. Smartwatch versus SPIMU Correlation Results

#### 3.1.1. AP Correlation Results

**Figure 4:**
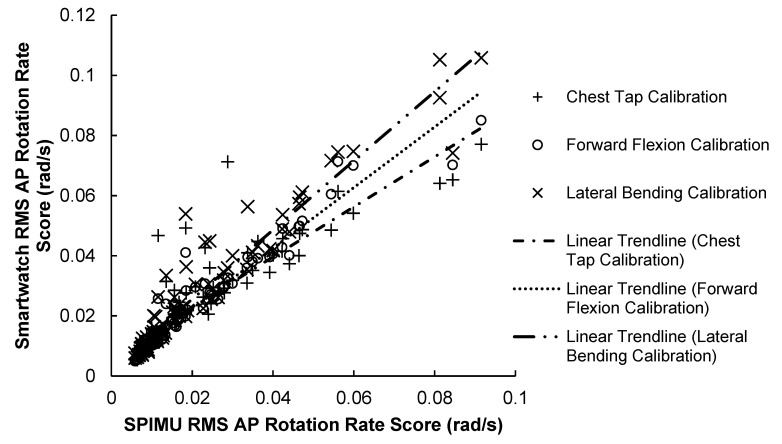
84 RMS AP rotation rate scores between smartwatch versus SPIMU. A positive trendline was produced with every calibration method.

The results of the AP scores correlation test are illustrated in Figure 4 and summarized in Table 2 below. Results show that the SPIMU and smartwatch AP scores for all calibration methods were strongly correlated across the 84 trials. Notably, the chest-tap calibration produced the weakest correlation (r = 0.884, *p* < 0.001): 7.8% weaker than the lateral bending maneuver (r = 0.953, *p* < 0.001) and 9.7% weaker than the forward flexion maneuver (r = 0.970, *p* < 0.001). The forward flexion calibration method produced a slightly stronger correlation compared with the lateral bending maneuver.

#### 3.1.2. ML Correlation Results

**Figure 5:**
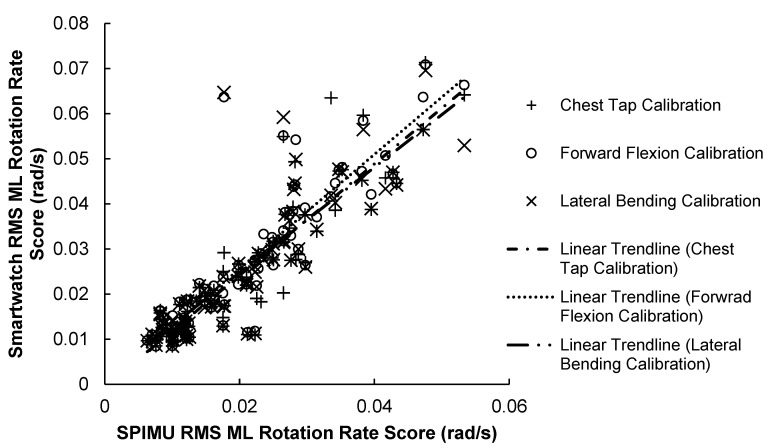
84 RMS ML rotation rate scores between smartwatch versus SPIMU. A positive trendline was produced.

The results of the ML scores correlation test are illustrated in Figure 5 and summarized in Table 3 below. Results show that the SPIMU and smartwatch ML scores were strongly correlated across the 84 trials. Notably, the chest-tap calibration produced the strongest correlation (r = 0.901, *p* < 0.001): 4.4% stronger than the lateral bending maneuver (r = 0.861, *p* < 0.001) and 1.5% stronger than the forward flexion maneuver (r = 0.887, *p* < 0.001). The forward flexion calibration method produced slightly stronger correlation compared with the lateral bending calibration method.

##### 3.1.3. 2D Correlation Results

**Figure 6:**
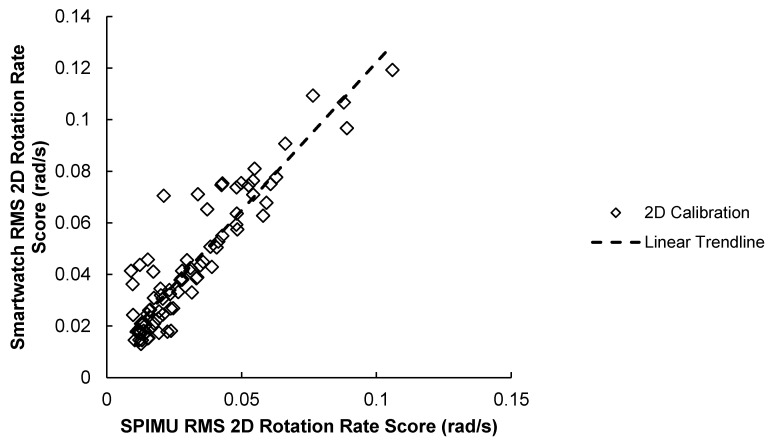
84 RMS 2D rotation rate scores between smartwatch versus SPIMU. A positive trendline was produced.

The results of the 2D scores correlation test are illustrated in Figure 6 and summarized in Table 4 below. Results show that the SPIMU and smartwatch 2D scores were strongly correlated across the 84 trials (r = 0.919, *p* < 0.001).

### 3.2. Smartwatch versus Force Plate Correlation Results

#### 3.2.1. AP Correlation Results

**Figure 7:**
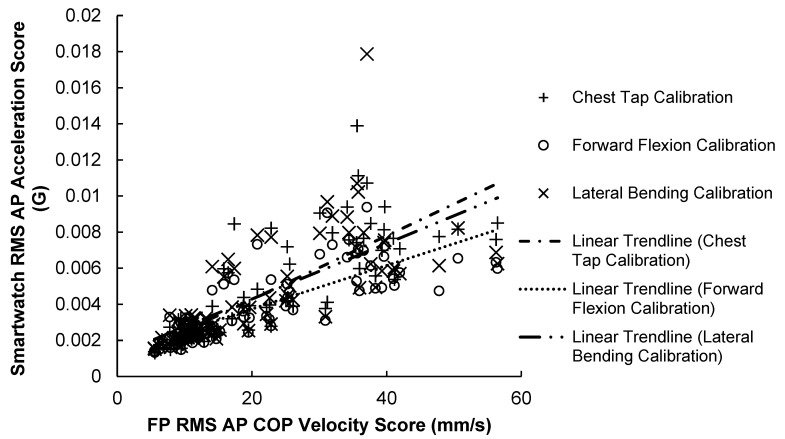
84 Smartwatch RMS AP acceleration scores versus force-plate RMS AP COP velocity scores. A positive trendline was produced with every calibration method.

The results of the AP scores correlation test are illustrated in Figure 7 and summarized in Table 5. Results show that AP scores for the force plate and smartwatch were strongly correlated (r = 0.721–0.819, *p* < 0.001) across the 84 trials. The SPIMU showed the weakest correlation (r = 0.281, *p* = 0.010) with the force plate, compared with all three smartwatch scores.

#### 3.2.2. ML Correlation Results

**Figure 8:**
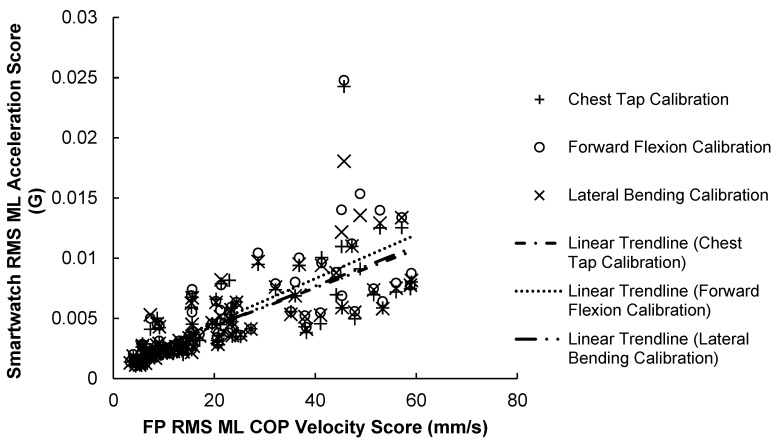
84 Smartwatch RMS ML acceleration scores versus force-plate RMS ML COP velocity scores. A positive trendline was produced with every calibration method.

The results of the ML scores correlation test are illustrated in Figure 8 and summarized in Table 6 below. Results show that ML scores for the force plate and smartwatch were strongly correlated (r = 0.729–0.799, *p* < 0.001) across the 84 trials. The SPIMU showed the weakest correlation with the force plate (r = 0.711, *p* < 0.001), compared with all three smartwatch scores.

##### 3.2.3. 2D Correlation Results

**Figure 9:**
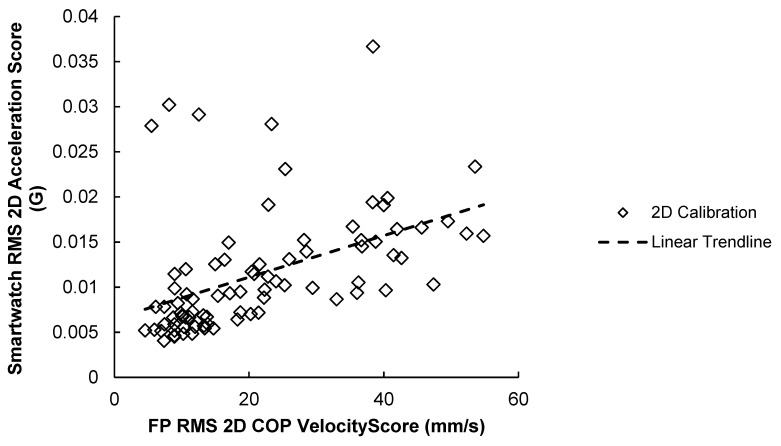
84 RMS 2D acceleration scores for smartwatch versus force-plate RMS 2D COP velocity scores. A positive trendline was produced.

The results of the 2D scores correlation test are illustrated in Figure 9 and summarized in Table 7. Results show that 2D scores for the force plate and smartwatch were moderately correlated across the 84 trials (r = 0.468, *p* < 0.001). This was the only posturography parameter where the SPIMU showed stronger correlation with the force plate (r = 0.593, *p* < 0.001) than the smartwatch.

### 3.3. Smartwatch Repeated-Measures Analysis of Variance Results

Sphericity was violated for several tests and indicated that only two of the three pose types produced significantly different scores. Results are described in detail in Section 3.3.1 and Section 3.3.2 below. As planned and stated in the methods section, to reduce type 1 error only the best SPIMU correlated calibration method was used in the ANOVA study; results from Section 3.1 show that the forward flexion maneuver had, on average, the strongest correlation with the SPIMU.

#### 3.3.1. RMANOVA Acceleration-Based Score Results

RMANOVA results for the pose-difficulty within-subject effects on IMU RMS acceleration scores and force-plate RMS COP velocity scores are shown in Table 8 below. Each direction was tested. The smartwatch and SPIMU revealed significant variation in the mean scores by pose type (*p* < 0.001) in the ML, 2D, and 3D directions, but not in the AP directions. The force plate also showed significant variation in the mean scores by pose type (*p* < 0.001) in all directions. Sphericity was violated in most cases, except for the 2D and 3D inertial scores (from both smartwatch and SPIMU).

#### 3.3.2. RMANOVA Rotational Velocity-Based Score Results

RMANOVA results for the within-subject effects of pose difficulty on RMS rotational velocity scores are shown in Table 9 below. Each direction was tested. The smartwatch found significant variation in the mean scores by pose type in the AP direction only—this direction did not show violation of sphericity. The 2D and 3D directions did not produce results within the corrected, experiment-wise significance level. For the SPIMU, all directions showed significant variation in the mean scores by pose type, and sphericity was violated for all cases except the ML direction. 

#### 3.3.3. Bar Graph and Standard Error Bars of the Smartwatch Scores

**Figure 10:**
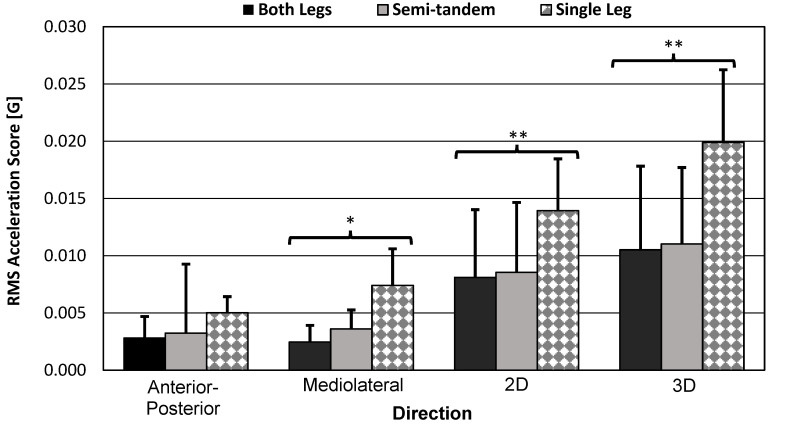
RMS acceleration score bar graphs. Bar graphs show sample mean; error bars indicate one standard deviation. Significance level was corrected to *α_e_* = 0.0026. RMANOVA test results were significant for ML (*p* < 0.001), 2D (*p* = 0.002), and 3D (*p* < 0.001) scores. * = significant variation in the mean scores by pose type. ** = significant equal variation in the mean scores by pose type.

**Figure 11:**
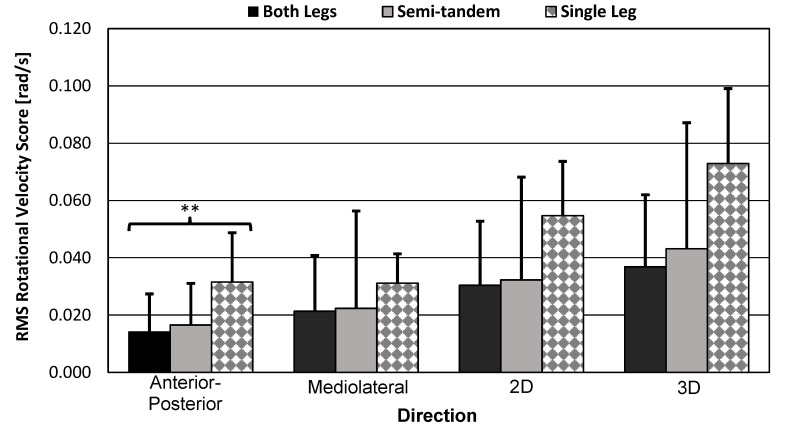
RMS rotational velocity score bar graphs. Bar graphs show sample mean; error bars indicate one standard deviation. Significance level was corrected *α_e_* = 0.0026. RMANOVA test results were significant for AP scores only (*p* < 0.001). ** = significant equal variation in the mean scores by pose type.

The smartwatch results for each pose type and for each direction are visualized in Figure 10 and Figure 11 below. The bar graphs themselves represent mean scores across participants. Within-subject variation in the mean scores by pose-type results are indicated with asterisks to summarize results of the previous RMANOVA tests. The results show that the scores for the both-legs and semi-tandem poses were similar, which may explain why sphericity was violated in many of the tests. 

#### 3.3.4. Bar Graph and Standard Error Bars of the Force-Plate Scores

**Figure 12:**
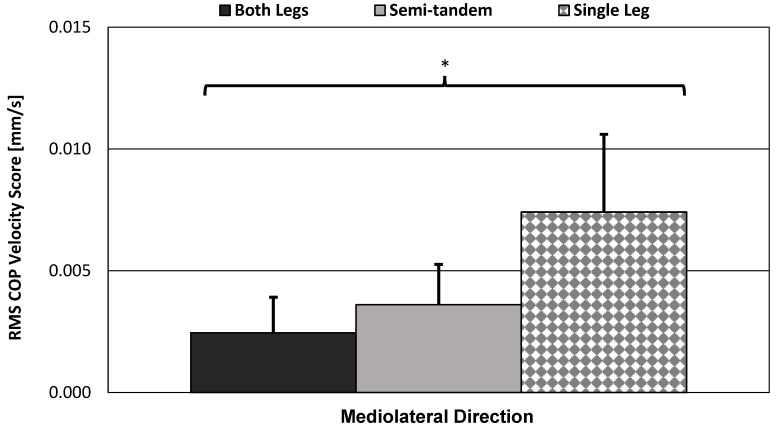
Force plate’s RMS ML COP velocity bar graphs. Bar graphs show sample mean; error bars indicate one standard deviation. Significance level was corrected to *α_e_* = 0.0026. RMANOVA results showed significant variation in the mean scores by pose type, but sphericity was violated even in best case (Factor DOF = 1.10, F = 83.23, *p* < 0.001). * = significant variation in the mean scores by pose type.

Since sphericity was violated in many cases, including the gold-standard force-plate method (see Table 8), it was appropriate to assess the bar graph and standard error bars of the poses. Figure 12 shows the force plate’s mean ML COP velocity scores, which were its best results for detecting equal variation in the mean scores by pose type (i.e., highest RMNAOVA factor DOF, pose-type difficulty increased linearly).

The single-leg stance produced much higher RMS scores. The small differences in difficulty between the both-legs and semi-tandem stances may be a good way to test the sensitivity of each of these devices. However, since the single leg was so much more difficult than these two stances, devices were rarely able to capture spherical variation in the mean scores by pose type (i.e., score variance was rarely equal between all three poses).

### 3.4. Qualitative Results

**Figure 13:**
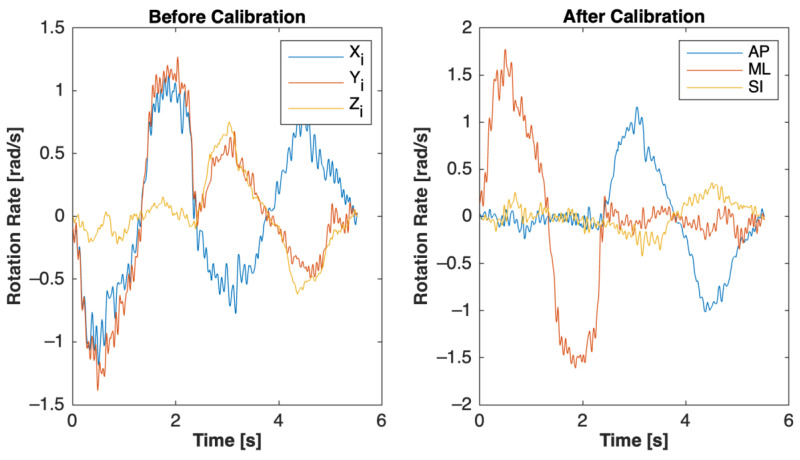
1 Sample of before and after calibration of smartwatch rotational movement. Rotation-based maneuvers are shown (i.e., first forward flexion and then lateral bending). The one-dimensional maneuvers in the global coordinate system after calibration can be clearly seen (i.e., first ML rotation then AP rotation), whereas in the inertial system, the maneuvers are an unobservable three-dimensional movement. The AP rotation of the lateral bending maneuver was more convoluted than the ML rotation of the forward flexion maneuver; apparent secondary and tertiary rotation occurred during this potentially more difficult calibration maneuver.

Functional calibration has not been pursued in posturography studies, so some additional calibration/alignment results are shown below. During the calibration development, many figures were developed that showed the algorithm’s ability to capture the global coordinate system. Figure 13 shows how the smartwatch projected data onto a global coordinate system during the calibration maneuvers. After calibration, there were clear movements in the ML and then the AP directions, reflecting the order in which the calibration maneuvers were performed.

## 4. Discussion

### 4.1. Summary of Key Findings

The smartwatch produced posturography scores that were strongly correlated with state-of-the-art methods for posturography. The smartwatch data’s strong correlation with the SPIMU data (r = 0.861–0.970, *p* < 0.001) indicates that the functional calibration algorithm was effective. The smartwatch was also moderately to strongly correlated with the force plate (r = 0.468–0.821, *p* < 0.001). In AP and ML acceleration scores, the smartwatch showed stronger correlation with the force plate than the SPIMU.

The smartwatch was able to detect significant variation in the mean scores by pose type in most of the acceleration metrics. Specifically, the smartwatch detected significant pose type effects for:

- Acceleration scores in the ML, 2D, and 3D directions.
- Rotational scores in the AP direction.

Significant variation in the mean scores by pose type supported the hypothesis that the smartwatch can detect differences in balance poses that increase in difficulty. Additional descriptive plots are illustrated, demonstrating significant or insignificant variation in the mean scores by pose type (Figure 10 and Figure 11). Additionally, the medium difficulty pose type (i.e., semi-tandem stance) introduced detectable ML instability only (i.e., increase in ML acceleration, increase in AP rotation). However, AP instability was too similar between the both-legs and semi-tandem stances, which probably affected the RMANOVA’s factor DOF.

Since 2D and 3D acceleration scores were also able to detect significant variation in the mean scores by pose type, this may indicate that anatomical calibration is not always necessary for pose-type detection. However, AP and ML directions should still be considered when conducting experiments on participants struggling with balance deficit. 

### 4.2. Limitations of RMANOVA Statistical Results

The force plate, which is considered the gold standard, violated the sphericity assumption across each direction. The both-legs and semi-tandem stances were too similar in difficulty to allow for equal variation in the mean scores by pose type (see Figure 12). Furthermore, the force plate’s degrees of freedom were modified to 1.03 through 1.10 (see Table 8)—indicating that the pose-type variable was only significantly different between two of the three poses (DOF = 1.03 (worst case), *p* < 0.001). 

Additionally, the sample size for the RMANOVA study was limited to 18 healthy participants. However, even with this smaller sample size and Bonferroni correction, significant results were found. The current statistical results can also be used to determine a sufficient number of subjects required to achieve adequate levels of power in future related studies. Section 4.4 details future work focusing on groups of individuals with balance deficit.

### 4.3. Optional Improvement to Calibration Algorithm

The algorithm can be easily adjusted to ensure three orthogonal target vectors. This is possible by removing the vertical target-vector components out of the PCA target vector (i.e., projecting the PCA target vector onto the plane defined by the vertical target vector). This ensures that there is no overlap of the two vectors (i.e., the dot product between the vectors is zero). This procedure is shown in the equation:

(19) $$v_{PCA_{mod}}=v_{PCA}-(v_{PCA}\cdot v^{SI})v^{SI}$$

This is the result of a common vector proof in mathematics. The $v_{PCA}$ is any target vector found with PCA before it has been scaled or checked for the correct sign. For example, this would be applied just before Equation (8) in the forward flexion calibration process. Recall, these vectors are unit vectors.

However, if the vertical target vector experienced an error during its capture, it may also create an error in the PCA vector and the resulting cross product-based vector [14]. Noise from the magnetic field was not found to be an issue in testing—despite being inside around other electronic equipment—although it remains a valid concern for functional calibration methods.

### 4.4. Future Development of IMU-Based Posturography

For future clinical situations inside and outside laboratory settings, routine measurements should be taken from participants with balance deficit to track RMS acceleration scores over time. A similar RMANOVA test can be run to see whether the smartwatch can detect variance before and after treatments for the same pose. The factor in the RMANOVA would become “time under treatment” rather than “pose type.” The pose can be a standing posture or a sitting posture, but it must remain consistent and must be feasible for the participant to perform. It would be most desirable to take measurements periodically throughout the treatment process.

Normalization of balance scores by bodyweight, height, BMI, and age has not been explored. These factors may affect a participant’s scores but were not discussed in previous literature nor this study and may be addressed in future studies. 

Based on results from this study, forward flexion is the best calibration maneuver to perform. In cases where the participant was unable to perform the forward flexion maneuver, the smartwatch was shown to produce significant 2D and 3D acceleration scores. The participant could also perform the chest-tap maneuver, or researchers could propose another 1D maneuver. Additionally, the functional calibration maneuver could be used in a smart device application that guides participants through arm placement, calibration maneuver, pose instructions, and test duration. The RMS acceleration scores showed more significant variation in the mean scores by pose type than did the RMS rotational velocity scores, so RMS acceleration should be used in smartwatch inertia-based posturography.

## Figures and Tables

**Table 1:** Third Axis Target found with Cross Product.

Calibration Method (Type of Motion)	Known Axes (Method of Discovery)	Axis Found by Cross Product	Equation
Forward flexion (rotational velocity)	SI axis (gravity vector) ML axis (PCA vector)	AP axis	$v^{AP}=v^{ML}\times v^{SI}$
Lateral bending (rotational velocity)	SI axis (gravity vector) AP axis (PCA vector)	ML axis	$v^{ML}=v^{SI}\times v^{AP}$
Chest tap (acceleration)	SI axis (gravity vector) AP axis (PCA vector)	ML axis	$v^{ML}=v^{SI}\times v^{AP}$

**Table 2:** Correlation Strength and Significance of RMS AP Rotation Rate Scores between Smartwatch and SPIMU. Significance level is *α* = 0.05.

		Smartwatch Score		
		Chest-Tap Calibration	Forward Flexion Calibration	Lateral Bending Calibration
**SPIMU score**	**Pearson correlation**	0.884	0.970	0.953
	***p*-value (two-tailed)**	<0.001	<0.001	<0.001
	**Number of Data Points**	84	84	84

**Table 3:** Correlation Strength and Significance of RMS ML Rotation Rate Scores between Smartwatch and SPIMU. Significance level is *α* = 0.05.

		Smartwatch Score		
		Chest-Tap Calibration	Forward Flexion Calibration	Lateral Bending Calibration
**SPIMU score**	**Pearson correlation**	0.901	0.887	0.861
	***p*-value** **(two-tailed)**	<0.001	<0.001	<0.001
	**Number of Data Points**	84	84	84

**Table 4:** Correlation Strength and Significance of RMS 2D Rotation Rate Scores between Smartwatch and SPIMU. Significance level is *α* = 0.05.

		Smartwatch 2D Score
		(2D Calibration Method)
**SPIMU score**	**Pearson correlation**	0.919
	***p*-value** **(two-tailed)**	<0.001
	**Number of Data Points**	84

**Table 5:** Correlation Strength and Significance of RMS AP Scores between Smartwatch and Force Plate. Posturography parameters for smartwatch and force-plate scores are acceleration and COP velocity, respectively. SPIMU results shown for comparison. Significance level is *α* = 0.05.

		Smartwatch Score			
		Chest-Tap Calibration	Forward Flexion Calibration	Lateral Bending Calibration	SPIMU
**Force-plate score**	**Pearson** **correlation**	0.819	0.794	0.721	0.281
	***p*-value (two-tailed)**	<0.001	<0.001	<0.001	0.010
	**Number of Data Points**	84	84	84	84

**Table 6:** Correlation Strength and Significance of RMS ML Scores between Smartwatch and Force Plate. Posturography parameters for smartwatch and force-plate scores are acceleration and COP velocity, respectively. SPIMU results shown for comparison. Significance level is *α* = 0.05.

		Smartwatch Score			
		Chest-Tap Calibration	Forward Flexion Calibration	Lateral Bending Calibration	SPIMU
**Force-plate score**	**Pearson** **correlation**	0.729	0.758	0.799	0.711
	***p*-value** **(two-tailed)**	<0.001	<0.001	<0.001	<0.001
	**Number of Data Points**	84	84	84	84

**Table 7:** Correlation Strength and Significance of RMS 2D Scores between Smartwatch and Force Plate. Posturography parameters for smartwatch and force-plate scores are acceleration and COP velocity, respectively. SPIMU results shown for comparison. Significance level is *α* = 0.05.

		Smartwatch 2D Score	SPIMU
		(2D Calibration Method)	
**Force-plate score**	**Pearson** **correlation**	0.468	0.593
	***p*-value** **(two-tailed)**	<0.001	<0.001
	**Number of Data Points**	84	84

**Table 8:** RMANOVA for Acceleration-based Posturography Scores. Uncorrected Factor DOF is two (i.e., there were three pose types). Uncorrected Error DOF is 34 (i.e., there were 18 participants). Significance level was corrected to *α_e_* = 0.0026.

Device	Direction	Pose-Type within-Subject Effects on RMS Score			
		Error DOF	Factor DOF	F	*p*-Value
Smartwatch (forward flexion)	AP	18.44	1.09	1.73	0.205
	ML	21.51	1.27	40.70 *	<0.001
	2D	34	2.00	8.08 **	0.002
	3D	34	2.00	16.06 **	<0.001
SPIMU	AP	34	2.00	3.31	0.048
	ML	19.10	1.12	20.43 *	<0.001
	2D	34	2.00	15.49 **	<0.001
	3D	34	2.00	17.16 **	<0.001
Force plate (COP Vel.)	AP	18.15	1.07	61.27 *	<0.001
	ML	18.62	1.10	83.23 *	<0.001
	2D	17.52	1.03	52.17 *	<0.001

* Indicates significant F statistic (i.e., variation in the mean scores by pose type). ** Indicates significant F statistic without violation of sphericity (i.e., equal variation in the mean scores by pose type).

**Table 9:** RMANOVA for Rotational Velocity-based Posturography Scores. Uncorrected Factor DOF is two (i.e., there were three pose types); Uncorrected Error DOF is 34 (i.e., there were 18 participants). Significance level was corrected to *α_e_* = 0.0026.

Device | Calibration Method	Direction	Pose-Type Within-Subject Effects			
		Error DOF	Factor DOF	F	*p*-Value
Smartwatch (forward flexion)	AP	34	2.00	9.59 **	<0.001
	ML	25.51	1.5	0.41	0.609
	2D	34	2.00	3.40	0.028
	3D	34	2.00	6.55	0.004
SPIMU	AP	20.59	1.21	24.74 *	<0.001
	ML	34	2.00	12.23 **	<0.001
	2D	23.64	1.39	23.33 *	<0.001
	3D	23.53	1.38	37.47 *	<0.001

* Indicates significant F statistic (i.e., variation in the mean scores by pose type). ** Indicates significant F statistic without violation of sphericity (i.e., equal variation in the mean scores by pose type).

## Data Availability

Interested parties can contact sklisch@calpoly.edu. Additionally, all data are available at California Polytechnic State University’s digital commons.
